# Environmental Public Health Dimensions of Shale and Tight Gas Development

**DOI:** 10.1289/ehp.1307866

**Published:** 2014-04-16

**Authors:** Seth B.C. Shonkoff, Jake Hays, Madelon L. Finkel

**Affiliations:** 1 Physicians Scientists and Engineers for Healthy Energy, Oakland, California, USA; 2 Department of Environmental Science, Policy, and Management, University of California, Berkeley, Berkeley, California, USA; 3 Physicians Scientists and Engineers for Healthy Energy, New York, New York, USA; 4 Department of Public Health, Weill Cornell Medical College, New York, New York, USA

## Abstract

Background: The United States has experienced a boom in natural gas production due to recent technological innovations that have enabled this resource to be produced from shale formations.

Objectives: We reviewed the body of evidence related to exposure pathways in order to evaluate the potential environmental public health impacts of shale gas development. We highlight what is currently known and identify data gaps and research limitations by addressing matters of toxicity, exposure pathways, air quality, and water quality.

Discussion: There is evidence of potential environmental public health risks associated with shale gas development. Several studies suggest that shale gas development contributes to ambient air concentrations of pollutants known to be associated with increased risk of morbidity and mortality. Similarly, an increasing body of studies suggest that water contamination risks exist through a variety of environmental pathways, most notably during wastewater transport and disposal, and via poor zonal isolation of gases and fluids due to structural integrity impairment of cement in gas wells.

Conclusion: Despite a growing body of evidence, data gaps persist. Most important, there is a need for more epidemiological studies to assess associations between risk factors, such as air and water pollution, and health outcomes among populations living in close proximity to shale gas operations.

Citation: Shonkoff SB, Hays J, Finkel ML. 2014. Environmental public health dimensions of shale and tight gas development. Environ Health Perspect 122:787–795; http://dx.doi.org/10.1289/ehp.1307866

## Introduction

Technological innovations in drilling and well-stimulation techniques have led to the production of natural gas from previously inaccessible geological formations, such as shale. Proponents of modern gas development argue that it has created a unique economic and political opportunity. Some in the public health community, however, have concerns about the potential for the extraction process to negatively impact the environment and human health (Finkel et al. 2013; Goldstein et al. 2012; Saberi 2013; Witter et al. 2013).

Producing natural gas from shale and tight gas formations in an economically feasible manner frequently requires a new constellation of existing technologies: high-volume, slickwater, hydraulic fracturing from clustered, multiwell pads using long directionally drilled laterals. This method can involve drilling a well vertically thousands of feet below the surface and then directionally (horizontally) for up to 2 miles. An average of 2–5 million gallons of fluid consisting of water, proppant (often crystalline silica), and chemicals (some of which are known carcinogens or otherwise toxic) are injected into the well at a pressure high enough to fracture the shale rock [U.S. Environmental Protection Agency (EPA) 2010a]. Chemicals often referred to as slickwater are added to the fracturing fluid to decrease its friction. The fracturing fluid creates and expands cracks in the shale. When the pressure is released, the cracks are held open by the sand, allowing the tightly held gases to flow into the cracks and up the production casing. The gas is then collected, processed, and sent through transmission pipelines to market. In 2012, shale gas constituted nearly 40% of U.S. gas production, up from 2% in 2000 (Hughes 2013).

Natural gas has a variety of attractive attributes. In the current market, it is a relatively inexpensive and abundant fuel. When combusted for electricity generation, it emits fewer health-damaging contaminants and approximately 50% less carbon dioxide emissions compared with burning coal (U.S. Energy Information Administration 2013). Yet, emerging scientific evidence suggests that there may be health risks associated with the development of shale gas.

In this review we discuss the body of scientific literature relevant to the environmental public health impacts of shale gas production. We highlight what is currently known and identify data gaps and research limitations.

## Methods

*Scope of review*. For this review, we focused primarily on literature directly pertinent to the human health dimensions of shale and tight gas development. “Tight gas” refers to natural gas produced from reservoir rocks of low permeability, such as shale or sandstone. Shale gas and other forms of tight gas are referred to as “unconventional” because of their atypical reservoirs, which require new production techniques. However, we cite some studies that did not directly evaluate unconventional natural gas operations, but that are nonetheless relevant to various aspects of the overall process [e.g., particulate matter (PM) pollution, ozone]. In the case of ozone, for instance, we analyzed top-down studies that measured tropospheric concentrations rather than studies that supplied bottom-up measurements (e.g., leakage rates). Publications included in our review are predominantly sourced from the peer-reviewed scientific literature but include, where appropriate, government reports and other gray literature. Although the production chain of gas development is far reaching, we focused on the processes that begin with trucking the water, sand, chemicals, and other materials to the well pad, and end with the disposal of wastewater. Evidence suggests that these processes present the greatest risks to environmental public health and therefore have received the most attention in the scientific literature (Korfmacher et al. 2013; McKenzie et al. 2012; Rozell and Reaven 2012; Witter et al. 2013).

*Terminology*. Terminology is important when discussing modern forms of natural gas development. In part because of a lack of well-defined, uniform terminology, there has been confusion regarding which processes constitute this type of development. The terms, “hydraulic fracturing” or “fracking” are regularly used in the popular media as umbrella terms to describe the entire process of obtaining shale gas, as well as other forms of unconventional natural gas development, from land clearing and well spudding to transmission of natural gas to market. However, taken literally, “hydraulic fracturing” refers only to well-stimulation processes and excludes other potentially more health and environmentally impactful processes, including but not limited to well drilling, fracturing-fluid production, wastewater disposal, transportation of materials, and the processing, compression, and transmission of gas and liquids.

Many of the studies we cite in this review may also apply to other forms of oil and gas development that use well-stimulation techniques, including matrix acid stimulation, acid fracturing, and steam injection. However, these other techniques are beyond the focus of this review. The term “unconventional oil and gas development” can also refer to bitumen/tar sands extraction and processing, and other types of fossil fuel development that employ novel engineering and production techniques to obtain fuels from unconventional resources (e.g., coalbed methane) that are beyond the scope of our review. Because most of the environmental public health–relevant scientific literature on modern oil and gas production has focused on the development of natural gas from shale formations, we use the term “shale gas development.” However, here we discuss, where appropriate, scientific literature on other forms of unconventional or tight gas development that include the most prominent and relevant features of shale gas development, such as high-volume, horizontal hydraulic fracturing.

*Identification of relevant studies*. The literature directly relevant to the environmental public health dimensions of shale gas development is still limited. For this reason, we adopted a broad search strategy comprising the following:

Systematic searches in three peer-reviewed science databases across multiple disciplines: PubMed (http://www.ncbi.nlm.nih.gov/pubmed/), Web of Science (http://www.webofknowledge.com), and ScienceDirect (http://www.sciencedirect.com)
Searches in existing collections of scientific literature on this subject, such as the Marcellus Shale Initiative Publications Database at Bucknell University (http://www.bucknell.edu/script/environmentalcenter/marcellus), complemented by Google (http://www.google.com) and Google Scholar (http://scholar.google.com)
Manual searches (hand-searches) of references included in all peer-reviewed studies that pertained directly to shale gas development.

For bibliographic databases, we used a combination of Medical Subject Headings (MeSH)-based and keyword strategies, which included the following terms, as well as relevant combinations:

shale gas, shale, hydraulic fracturing, fracking, drilling, natural gas production, Marcellus, Barnett, Denver-Julesberg Basin, air pollution, methane, water pollution, public health, water contamination, fugitive emissions, air quality, epidemiology, unconventional gas development, and environmental pathways.

This search identified a total of 211 peer-reviewed publications that pertain directly to shale gas development. [This database, the PSE STUDY CITATION DATABASE on Shale Gas & Tight Oil Development, is available online (http://psehealthyenergy.org/site/view/1180), and we will continue to update it with relevant literature.] Of these 211 publications, only 33 presented original data that met our inclusion criteria and that we considered relevant as primary literature.

*Inclusion/exclusion criteria*. From the studies identified through 1 February 2014, we excluded nonrelevant technical papers and studies related to economics, climate change, sociology, regulation, seismicity, water usage, social stress, and quality of life considerations. Although we excluded commentaries from the results of our review, a few are cited in order to provide documentation of particular considerations among the public health community. We included studies with direct pertinence to the environmental public health and environmental exposure pathways (i.e., air and water) associated with shale and tight gas development. In this regard, we supplemented the shale gas literature with studies that evaluated particular environmental pathways and health outcomes. For instance, we included studies directly related to the health impacts of tropospheric ozone, fine particulate air pollution, and endocrine-disrupting chemicals (EDCs). We excluded the vast majority of non–peer-reviewed scientific literature, but environmental impact statements and other government reports are cited where appropriate.

## Results

**Figure 1:**
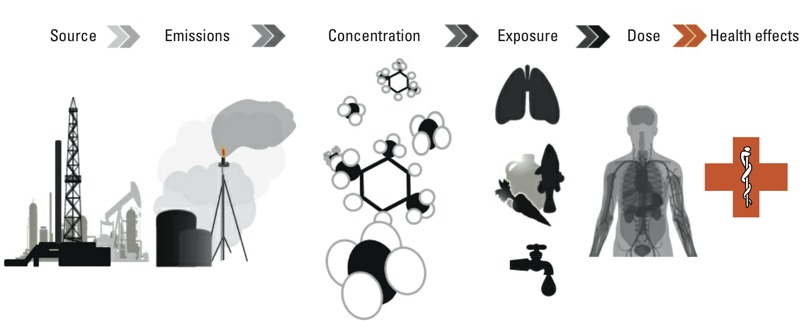
The environmental exposure pathway provides an analytical framework to describe, in broad terms, the connections between pollutant sources and human health outcomes. This framework begins with the emission source, in this case a well pad and associated infrastructure, which emit a variety of contaminants into the air, water, and soil. The concentrations of pollutants in the air, water, and soil that result from these emissions influence the magnitude of human exposures through organs such as the nose, mouth, and skin. Once the level of exposure is identified, it is then possible to estimate the dose, or how much of the pollutant is ingested in a given period of time. The dose, in turn, determines the health outcome.

*The environmental public health framework and possible exposure pathways*. The environmental exposure pathway framework is often used to describe associations between pollutant sources and health effects via emissions, environmental concentrations of pollutants, pollutant exposure pathways (e.g., mouth, nose, ears, eyes, skin), and dose (i.e., micrograms of pollutant ingested per day) (Figure 1) (Agency for Toxic Substances and Disease Registry 2005).

Potential sources of health-relevant environmental pollution are present throughout many phases of shale gas development. These sources include shale gas production and processing activities (i.e., drilling, hydraulic fracturing, hydrocarbon processing and production, and wastewater disposal); the transmission and distribution of the gas to market (i.e., transmission lines and distribution pipes); and the transportation of water, sand, chemicals, and wastewater before, during, and after hydraulic fracturing.

## Hydraulic Fracturing Fluids: Chemical Toxicology and Exposure Pathways

Shale gas development uses fracturing fluids that contain organic and inorganic chemicals known to be health damaging (Aminto and Olson 2012; U.S. House of Representatives, Committee on Energy and Commerce 2011). Fracturing fluids can move through the environment and come into contact with humans in a number of ways, including surface leaks, spills, releases from holding tanks, poor well construction, leaks and accidents during transportation of fluids, flowback and produced water to and from the well pad, and run-off during blowouts, storms, and flooding events (Rozell and Reaven 2012). Further, the mixing of these compounds under conditions of high pressure—and often high heat—may synergistically create additional potentially toxic compounds (Kortenkamp et al. 2007; Teuschler and Hertzberg 1995; Wilkinson et al. 2000). Compounds found in these mixtures may pose risks to the environment and to public health through numerous environmental pathways, including water, air, and soil (Leenheer et al. 1982).

Chemicals are used in drilling and fracturing processes as corrosion inhibitors, biocides, surfactants, friction reducers, gels, and scale inhibitors, among others (Aminto and Olson 2012; New York State Department of Environmental Conservation 2011; Southwest Energy 2012). These chemicals include methanol, ethylene glycol, naphthalene, xylene, toluene, ethylbenzene, formaldehyde, and sulfuric acid, some of which are known to be toxic, carcinogenic, or associated with reproductive harm (Colborn et al. 2011; New York State Department of Environmental Conservation 2011). Many of these compounds are considered hazardous water pollutants and are regulated in other industries (Clean Water Act of 1972; Safe Drinking Water Act of 1974; U.S. House of Representatives 2011).

Many of the chemical compounds used in the fracturing process lack scientifically based maximum contaminant levels, making it more difficult to quantify their public health risks (Colborn et al. 2011). Moreover, uncertainty about the chemical makeup of fracturing fluids persists because of the limitations on required chemical disclosure, driven by the Energy Policy Act of 2005. For instance, in many states, companies are not mandated to disclose information about the quantities, concentrations, or identities of chemicals used in the process on the principle that trade secrets might be revealed (Centner 2013; Centner and O’Connell 2014; Maule et al. 2013).

Some companies make efforts to be more transparent in the disclosure of chemicals used in the process. FracFocus (http://www.fracfocus.org) was developed as an online, voluntary chemical disclosure registry, and some agencies (e.g., Bureau of Land Management) have suggested that it be used as a regulatory compliance tool (Konschnik et al. 2013). However, the registry has been criticized because of uncertainty surrounding the timing, substance, and omissions of the disclosed data on the website (Konschnik et al. 2013).

Because of the limited information that is available, researchers have sought to acquire more information on the chemical makeup of fracturing fluids through other means. For example, using material safety data sheets, Colborn et al. (2011) identified 353 of 632 chemicals contained in 944 products used for natural gas operations in Colorado, and they examined available information on each product. Their study represents one of the first attempts to conduct a chemical hazard assessment by identifying some of the compounds in fracturing fluids.

It should be noted that the scope of the study by Colborn et al. (2011) is limited in that they did not measure exposure, dose, or health outcomes across populations. The researchers identified Chemical Abstract Service (CAS) numbers for the chemicals and used these in systematic searches of databases such as TOXNET (http://toxnet.nlm.nih.gov). On the basis of search results, the researchers classified the compounds into 12 health-effects categories. At certain concentrations or doses, > 75% of the chemicals they identified are known to negatively impact the skin, eyes, and other sensory organs; the respiratory system; the gastrointestinal system; and the liver. Fifty-two percent of the chemicals have the potential to negatively affect the nervous system, and 37% are candidate EDCs (Colborn et al. 2011).

EDCs present unique hazards, particularly during fetal and early childhood growth and development (Diamanti-Kandarakis et al. 2009). They can affect the reproductive system and epigenetic mechanisms that may lead to pathology decades after exposure (Zoeller et al. 2012). EDCs have challenged traditional concepts in toxicology because effects at higher doses do not always predict effects at low doses (Vandenberg et al. 2012). In other words, the dose does not always make the poison.

Kassotis et al. (2014) measured estrogen and androgen receptor activity in surface and groundwater samples in Colorado using reporter gene assays in human cell lines. Water samples collected from the more intensive areas of natural gas development exhibited statistically significantly more estrogenic, antiestrogenic, or antiandrogenic activity than references sites with either no operations or fewer operations (Kassotis et al. 2014). The concentrations of chemicals detected were in high enough concentrations to interfere with the response of human cells to male sex hormones and estrogen. This study by Kassotis et al. (2014) indicated that EDCs are a potential health concern in natural gas operations, and suggested that chemicals used in the process should be screened for EDC activity.

## Air Quality

Air pollutant emission sources from shale gas development can be grouped into two main categories: *a*) emissions from drilling, processing, well completions, servicing, and other gas production activities; and *b*) emissions from transportation of water, sand, chemicals, and equipment to and from the well pad.

*Air pollution: drilling, well stimulation, gas production, processing, and servicing*. The literature suggests that shale gas development processes emit hazardous air pollutants including—but not limited to—BTEX compounds (benzene, toluene, ethylbenzene, and xylene), formaldehyde, hydrogen sulfide, acrylonitrile, methylene chloride, sulfuric oxide, nitrogen oxides (NO_x_), volatile organic compounds (VOCs), trimethylbenzenes, aliphatic hydrocarbons, diesel PM, and radon gas (McKenzie et al. 2012; Pétron et al. 2012; Roy et al. 2013). These emissions can result in elevated air pollution concentrations that exceed U.S. EPA guidelines for both carcinogenic and noncarcinogenic health risks (McKenzie et al. 2012; Meteorological Solutions Inc. 2011).

McKenzie et al. (2012) used U.S. EPA guidance to estimate chronic and subchronic non-cancer hazard indices (HIs) and cancer risks from exposure to hydrocarbons for residents living > 0.5 mile and ≤ 0.5 mile from wells in Colorado. The authors found that residents living ≤ 0.5 mile from wells were at a greater risk for health effects from exposure to natural gas development than those living > 0.5 mile from wells. Notably, they found a subchronic non-cancer HI of 5 for those living ≤ 0.5 mile compared with an HI of 0.2 for those living > 0.5 mile from wells, which was driven primarily from exposure to trimethylbenzenes, xylenes, and aliphatic hydrocarbons (McKenzie et al. 2012). Unfortunately, baseline air quality data prior to the study were not available. However, the statistically significant spatial associations between air quality and shale gas development indicate that air quality may be negatively impacted and health risks may increase during various stages of shale gas development.

Bunch et al. (2013), however, found that shale gas production activities did not result in community-wide exposures to concentrations of VOCs at levels that would pose a health concern. They compared VOC concentration data from seven air monitors at six locations in the Barnett Shale region in Texas with federal and state health-based air comparison values (HBACVs) in order to determine possible acute and chronic health effects; none of the concentrations exceeded acute HBACVs (Bunch et al. 2013). Air quality data included in their study were generated from monitors focused on regional atmospheric concentrations of pollutants (Bunch et al. 2013). Conversely, McKenzie et al. (2012) included samples at the community level in close proximity to gas development. Finer geographically scaled samples often capture local atmospheric concentrations that are more relevant to human exposure. This may be a primary reason why health hazard estimates differed between the two studies.

Roy et al. (2013) estimated emissions of NO_x_, VOCs, and PM for an air emissions inventory for the development of natural gas in the Marcellus Shale region for 2009 and 2020. They predicted that, in 2020, shale gas development activities would contribute 6–20% (mean, 12%) of the NO_x_ emissions and 6–31% (mean, 12%) of anthropgenic VOC emissions in Pennsylvania. However, these estimates were based on assumptions of improvements in gas production, completion, and processing infrastructure. If source-level emissions remain the same as in 2009, Marcellus VOC emissions were predicted to constitute approximately 34% (19–62%) of the regional anthropogenic VOC emissions in 2020 (Roy et al. 2013). Increases in emissions of VOCs and NOx, which are precursors of tropospheric ozone formation, could complicate ozone management in the region and potetentially offset ozone precursor emission reductions in other sectors at a time when several regions in Pennsylvania struggle to be within ozone attainment (Roy et al. 2013).

In another study focused on hydrocarbon emissions, Colborn et al. (2014) assessed air quality in western Colorado using weekly air samples collected before, during, and after drilling and hydraulic fracturing on a new natural gas well pad. They found numerous chemicals in the air samples that are associated with natural gas development operations, most notably methane, ethane, propane, and other alkanes. Many non-methane hydrocarbons (NMHCs), which were observed during the initial drilling phase, are associated with multiple health effects. Notably, 30 of the NMHCs they observed in the field were EDCs. In addition to the direct air pollution associated with natural gas drilling and processing (e.g., NMHCs, VOCs) outlined above, there are also indirect pollution concerns, such as the secondary atmospheric formation of tropospheric (ground-level) ozone (Colborn et al. 2014).

Studies have indicated that shale gas development is associated with the production of secondary pollutants such as tropospheric (ground-level) ozone, which is formed through the interaction of methane, VOCs, and NO_x_ in the presence of sunlight (Jerrett et al. 2009; U.S. EPA 2013). Tropospheric ozone is a strong respiratory irritant associated with increased respiratory and cardiovascular morbidity and mortality (Jerrett et al. 2009; United Nations Environment Programme 2011). Although toxicological data suggest that pure methane is not by itself health damaging (excluding its role as an asphyxiant and an explosive), it is a precursor to global tropospheric ozone (Smith et al. 2009).

Pétron et al. (2012) analyzed data collected at the National Oceanic and Atmospheric Administration (NOAA) Boulder Atmospheric Observatory (http://www.esrl.noaa.gov/psd/technology/bao) and filtered by wind sector, which indicated a high alkane and benzene signature from the direction of the Denver-Julesburg Basin, an area of considerable oil and gas development. The researchers found that an estimated 4% (range, 2.3–7.7%) of all natural gas (composed mostly of methane) produced was being accidentally leaked or purposefully vented to the atmosphere (Pétron et al. 2012). Karion et al. (2013) observed significant methane leaks in the Uintah Basin shale gas field, with an estimated 6.2–11.7% of total gas production leaking into the atmosphere.

In a national methane emissions study that combined ground and aerial sampling of methane with computer modeling, Miller et al. (2013) found that atmospheric levels of methane due to oil and gas extraction could be 4.9 ± 2.6 times greater than current estimates from the Emissions Database for Global Atmospheric Research (EDGAR) (http://edgar.jrc.ec.europa.eu/index.php) and the U.S. EPA. Although it is difficult to distinguish the sources of methane between oil and gas production and gas production, transmission, and storage, Peischl et al. (2013) estimated that 17% of gross methane production from oil and gas activities in the Los Angeles Basin are leaked or vented to the atmosphere.

Some studies have modeled ozone impacts associated with shale gas operations. Kemball-Cook et al. (2010) modeled ozone precursor emissions (VOCs and NO_x_) in the Haynesville Shale play that lies beneath the northeast Texas/northwest Louisiana border. Photochemical modeling for 2012 showed increases in 8-hr ozone design values of up to 5 ppb, which, along with the amount of projected emissions, give cause for concern about future atmospheric concentrations of ozone in Texas and Louisiana (Kemball-Cook et al. 2010). Olaguer (2012) used the Houston Advanced Research Center (HARC) neighborhood air quality model to simulate ozone formation near a hypothetical natural gas–processing facility, using estimates based on both regular and nonroutine (e.g. flaring) emissions. This model predicted that under average conditions, using regular emissions associated with compressor engines may significantly increase ambient ozone in the Barnett Shale formation (> 3ppb at 2 km downwind from the facility) (Olaguer 2012).

Substantial air quality impacts from oil and natural gas operations in Wyoming, Colorado, Utah, and Texas have also been directly measured (Carter and Seinfeld 2012; Edwards et al. 2013; U.S. Department of Energy 2011). Schnell et al. (2009) studied air quality in the rural Upper Green River Basin (UGRB) of Wyoming near the Jonah-Pinedale Anticline natural gas field in February 2008. They observed high photochemical ozone concentrations in the UGRB in the winter, reporting readings of up to 140 ppb, just less than double the U.S. EPA ozone concentration limit of 75 ppb (U.S. EPA 2012a). Before 2005, typical wintertime ozone concentrations in this area were 30–40 ppb (Pinto 2009). This increase in ozone concentration during this time period could be associated with the increase in NO_x_ and VOC emissions from oil and gas development activities in the area (Schnell et al. 2009). In a study conducted for the Wyoming Department of Environmental Quality, Meteorological Solutions Inc. (2011) found that the 8-hr ozone concentrations in the UGRB in 2011 exceeded the U.S. EPA ozone 8-hr standard for 13 days (Meteorological Solutions Inc. 2011) and exceeded the U.S. EPA scientists-recommended limit of 65 ppb for 25 days (Weinhold 2008).

In Utah there were 68 days in the winter of 2010 when ozone levels exceeded the U.S. EPA ozone standard of 75 ppb, and in 2011 there were readings more than double the U.S. EPA standard (Utah Department of Environmental Quality 2013). Results of experiments conducted by the U.S. EPA and NOAA indicated that ozone precursor emissions (VOCs and NO_x_, primarily) from oil and gas development in the Uintah Basin in Utah were a primary factor in the increased ozone level (Utah Department of Environmental Quality 2013).

Crystalline silica sand, used as a proppant (to “prop” open cracks in the target formation to allow gas to flow up the well), is delivered by trucks to the drilling site. Transporting this sand in trucks and trains and mixing it into fracturing fluids with sand movers, conveyer belts, and blender hoppers at the well site release silica dust into the air, where well-pad workers can be exposed (Esswein et al. 2013). Workers experience the most direct exposure; however, silica dust may also be an air contaminant of concern to nearby residents. The etiological association between respiratory exposure to silica dust and the development of silicosis is well known [Centers for Disease Control and Prevention (CDC) 1992, 2002]. Silicosis is a progressive lung disease in which tissue in the lungs reacts to silica particles, and can result in inflammation and scarring, which decreases the ability of the lungs to take in oxygen (CDC 1992, 2002). Respiratory exposure to silica is also associated with other diseases such as chronic obstructive pulmonary disease, tuberculosis, kidney disease, autoimmune conditions, and lung cancer (CDC 2002).

In cooperation with industry partners, Esswein et al. (2013) collected full-shift air samples at 11 sites in five states to determine levels of worker exposure. Of 111 air samples, 51.4% showed silica exposures greater than the calculated Occupational Safety and Health Administration permissible exposure level and 68.5% showed exposures greater than the National Institute for Occupational Safety and Health recommended exposure limit of 0.05 mg/m^3^ (Esswein et al. 2013). Further, these researchers noted that the type of respirators worn by workers were not sufficiently protective in some cases, given the magnitude of silica concentrations (Esswein et al. 2013).

*Air pollution: transportation*. Each well requires on average between 2 and 5 million gallons of water per hydraulic fracturing event (U.S. EPA 2010a). Water is generally not pumped directly to wells but is instead transported by diesel trucks, each of which has an approximate capacity of 3,000 gallons (U.S. EPA 2011b). It has been estimated that approximately 2,300 trips by heavy-duty trucks are required for each horizontal well during early stages of shale gas development (New York State Department of Environmental Conservation 2011). With thousands of such wells concentrated in high-development regions, levels of truck traffic and diesel-associated air pollution will increase in these areas.

The pollutant of primary health concern emitted from the transportation component of shale gas development is fine diesel PM. Diesel PM is a well-understood health-damaging pollutant that contributes to cardiovascular illnesses, respiratory diseases (e.g., lung cancer) (Garshick et al. 2008), atherosclerosis, and premature death (Pope 2002; Pope et al. 2004). For example, a study from the California Air Resources Board (Tran et al. 2008) indicated that there is an expected 10% (uncertainty interval: 3%, 20%) increase in the number of premature deaths per 10-μg/m^3^ increase in PM_2.5_ (PM ≤ 2.5 μm in aerodynamic diameter) exposure. Particulates can also contain concentrated associated products of incomplete combustion, and when particle diameter is < 2.5 μm, they can act as a delivery system to the alveoli of the human lung (Smith et al. 2009). In addition to diesel PM, as previously mentioned, NO_x_ and VOCs—other pollutants prevalent in diesel emissions—react in the presence of sunlight and high temperatures to produce tropospheric (ground-level) ozone.

## Water Quality

Rozell and Reaven (2012) conducted a risk assessment that identified five main pathways of water contamination in the shale gas production process: *a*) transportation spills of fracturing fluid or produced water; *b*) well casing leaks; *c*) leaks through fractured rock; *d*) drilling site discharge; and *e*) wastewater disposal. They found that wastewater disposal carries a potential risk of water contamination several orders of magnitude larger than that of the other pathways (Rozell and Reaven 2012).

Other studies have suggested that structural impairment of cement used to prevent transzonal gas migration in the wellbore is the most common mechanism through which groundwater can become contaminated (Vidic et al. 2013). Indeed, state environmental regulators at the Pennsylvania Department of Environmental Protection found that oil and gas development was responsible for polluting water supplies for at least 161 residences in Pennsylvania between 2008 and 2012, primarily due to cement structural integrity in wells and wellbores (Legere 2013). For the purpose of this review, we focused primarily on well casing leaks, drilling site discharge, and wastewater disposal because these are generally regarded as the most viable means of water contamination (Rozell and Reaven 2012; Vidic et al. 2013).

*Flowback and produced water*. Estimates of the proportion of fracturing fluid that returns to the surface as flowback and produced waters range from 9% to 80%, with most estimates around 35% (Horn 2009; New York State Department of Environmental Conservation 2011; U.S. EPA 2010a). These wastewaters contain the chemicals used in the fracturing fluid as well as compounds found deep in geological strata, such as salts, chlorides, heavy metals (e.g., cadmium, lead, arsenic), organic chemicals (e.g., BTEX compounds), bromide, and—depending on the geology—naturally occurring radioactive materials (e.g., radium-226). Many of these naturally occurring compounds are associated with human health effects when exposure is sufficiently elevated (Balaba and Smart 2012; Colborn et al. 2011; Haluszczak et al. 2013). A proportion of flowback and produced waters are treated and released as effluent or for other beneficial uses, such as irrigation for agriculture. However, many of the chemicals persist in high quantities because treatment facilities are unable to screen for and eliminate the complex array of compounds and products of synergistic interactions among them (Ferrar et al. 2013; Hladik et al. 2014; Lutz et al. 2013).

Flowback and produced water are sometimes treated at facilities and then discharged into surface waters (Ferrar et al. 2013). Warner et al. (2013a) examined water quality and isotopic compositions of discharged effluents, surface waters, and stream sediments associated with a Marcellus wastewater treatment facility site. Their findings suggest that insufficiently treated flowback and produced water that contained elevated concentrations of contaminants associated with shale gas development entered local water supplies, even after treatment. They also found elevated levels of chloride and bromide downstream, along with radium-226 levels in stream sediments at the point of discharge, that were approximately 200 times greater than upstream and background sediments and well above regulatory standards (Warner et al. 2013a). These types of water emissions may increase the health risks of residents who rely on these surface and hydrologically contiguous groundwater sources for drinking water (Wilson and VanBriesen 2012) and sources of food (i.e., fish protein) (Papoulias and Velasco 2013).

In a meta-analysis of chemical and physical characterizations of produced waters from shale gas, Alley et al. (2011) found that most of the produced waters generated by shale gas development were classified as saline (> 30,000 mg/L) or hypersaline (> 40,000 mg/L). These authors pointed out that treatment of this produced water for beneficial use often involves reverse osmosis, a practice that may generate a waste stream too large to justify the activity. Alley et al. (2011) also found that prior to treatment, produced waters can exceed toxicity thresholds of contaminants of concern, including—but not limited to—phosphates, cadmium, aluminum, barium, chloride, strontium, radium-226, bromine, lithium, and magnesium. Toxicity thresholds used in their meta-analysis were LC_50_ values (concentration lethal to 50%) for *Ceriodaphnia dubia* Richard, *Daphnia magna* Straus, and *Pimephales promelas* Rafinesque, and water-use criteria from the Food and Agricultural Organization of the United Nations guidelines for agricultural uses and the U.S. EPA Water Quality Criteria for surface discharge (Alley et al. 2011).

The results of Alley et al. (2011) agree with other reports that samples of fracturing fluids, drilling muds, and flowback and produced waters in wastewater–surface containment ponds contain chemicals that, at elevated doses or certain concentrations, have been associated with health effects ranging from skin and eye irritation to neurological and nervous system damage, cancer, and endocrine disruption (Colborn et al. 2011). Moreover, between July 2009 and June 2010, 192.5 million gallons of produced water was reported in Pennsylvania alone, with no certainty as to the location and type of disposal to be employed (Pennsylvania Department of Environmental Protection 2010).

The handling and disposal of flowback and produced water also hold implications for air quality because of volatile compounds, such as BTEX compounds, that are often mixed with the fluids. This may be particularly relevant when wastewater is stored in surface containment ponds and misted into the air to promote evaporation (Colborn et al. 2011).

*Gas and fluid migration*. Subsurface gas and fluid migration is most commonly associated with impaired structural integrity of well cement and, to a lesser extent, well casings. Failures in well barriers may allow intrusion of gases and fluids from producing formations below the casing shoe or from shallower gas- and fluid-bearing formations intersected by the wellbore to lower-pressure annuli. This may result in annular gas flow or sustained casing pressure and thus become a pathway for gas migration to the surface, a known mechanism of emissions of gases to the air and migration of gases and fluids to groundwater (Brufatto et al. 2003; Watson and Bachu 2009). Methane and other hydrocarbons can also migrate along improperly plugged wells, through an inadequately sealed annulus, or between geological zones as a result of cement failures in the wellbore (Vidic et al. 2013).

Leaking oil and gas wells have been recognized as a potential mechanism of subsurface migration of methane and heavier *n*-alkanes and other non-methane VOCs into groundwater and the atmosphere, contributing risks to drinking water and air quality (Bourgoyne et al. 2000; Brufatto et al. 2003; Chilingar and Endres 2005; Watson and Bachu 2009). Cement failures in onshore and offshore wells, reported to occur in 2–50% of all wells, provide pathways for gas migration to occur in the wellbore (Bourgoyne et al. 2000; Brufatto et al. 2003; Watson and Bachu 2009).

Because methane has a low solubility (26 mg/L at 1 atm, 20°C) (Vidic et al. 2013) and is relatively unreactive compared with longer-chain and unsaturated hydrocarbons (Jackson et al. 2011), it is typically regarded as nontoxic and is not regulated in the United States as a solute in water wells. However, there are no peer-reviewed studies on the health effects of chronic exposure to lower concentrations of methane in drinking water or indoor or outdoor air (Jackson et al. 2011). Further, if there is a pathway for methane migration, there could be a pathway for associated health-damaging gases coproduced with methane.

Some attention has been paid to the flammability of methane, the risk of explosions, and the risk of asphyxiation (primarily in high indoor concentrations). For example, in 2007 in Geauga County near Cleveland, Ohio, methane contaminated a water well and a home exploded; the Ohio Department of Natural Resources blamed a faulty concrete casing in a nearby gas well (Ohio Department of Natural Resources 2008). Similarly, in Pavillion, Wyoming, high concentrations of methane found in drinking water wells were attributed to gas production activities (DiGiulio et al. 2011). In addition, the U.S. EPA concluded that methane from geological layers not targeted for gas production migrated up the wellbore to an aquifer as a result of well cement failures in Parker County, Texas (U.S. EPA 2010b).

In certain regions, methane can naturally occur in aquifers, and there are conflicting scientific opinions about whether its presence is caused or exacerbated by shale gas development (Davies 2011; Saba and Orzechowski 2011; Schon 2011). However, there are convincing findings that shed light on the likelihood that shale gas development is associated with high methane levels in drinking water wells. Osborn et al. (2011) found that communities in Pennsylvania that had active shale gas development (one or more gas wells within 1 km) had statistically significantly higher concentrations of methane in their water wells compared with nonextraction sites (no shale gas wells within 1 km). The chemical signature of the methane found in drinking water wells in the active area indicated that the methane came from a high-pressure, deep-earth source (thermogenic methane). Alternatively, the methane from nonactive sites had signatures of shallow earth origins (biogenic methane). This suggests that shale gas production processes were the source of the methane contamination (Osborn et al. 2011).

Building on previous work by Osborn et al. (2011), Jackson et al. (2013) analyzed 141 drinking water wells across northeastern Pennsylvania. The researchers found methane in 82% of the samples (115 of 141 wells), with average concentrations six times higher for homes that were < 1 km from natural gas wells (59 of 141). These data, based on isotopic signatures and gas ratios, suggest that a subset of homeowners living < 1 km from shale gas wells had drinking water that was contaminated with stray gases associated with gas development activities (Jackson et al. 2013).

There is evidence that, in some locations, pathways exist between deep underlying formations and shallow drinking water aquifers (Vengosh et al. 2013). A modeling study by Myers (2012) suggested that pathways would allow for the transport of contaminants from the fractured shale to aquifers. Warner et al. (2012) found evidence of possible migration of Marcellus brine through naturally occurring pathways, based on strong geochemical fingerprints in salinized groundwater samples.

Both of these studies (Myers 2012; Warner et al. 2012) suggest that migration through fractured rock can serve as a subsurface contamination pathway to underground sources of drinking water. They also highlight the significance of the specific geographic configuration because some shallow drinking water resources are at more risk for contamination than others. In a study of the Fayetteville Shale in Arkansas, Warner et al. (2013b) suggested that methane contamination of shallow groundwater may not be a problem in certain shale formations. This difference may be attributed to geological variations across geographic space, including the presence of intermediate gas-bearing formations that are found overlying parts of some shale plays (e.g., Marcellus) but not others (e.g., Fayetteville).

In addition, Fontenot et al. (2013) evaluated water quality in private drinking water wells near natural gas operations in the Barnett Shale formation in Texas and found higher levels of arsenic, selenium, strontium, and total dissolved solids in wells located within 3 km of active gas wells. The authors used historical data from the region as a baseline to determine the contamination rates before the expansion of natural gas operations. Although heavy metals were present at low levels in aquifers in the region, concentrations were significantly higher in areas of active development (Fontenot et al. 2013). The authors were able to link contamination to natural gas activities; however, the specific factor responsible for contamination (e.g., well casing failures, mobilization of natural constituents, hydrogeochemical changes from lowering the water table) was not determined (Fontenot et al. 2013).

Researchers have been challenged in their ability to link associations between water contamination and unconventional natural gas development to any particular part of the process. After complaints about the taste and odor of well water from residents of Pavillion, Wyoming, the U.S. EPA initiated a groundwater investigation (DiGiulio et al. 2011). The observed water wells were located in an area known as the Pavillion gas field, which contained 169 gas production wells and 33 containment ponds used for storage/disposal of drilling wastes and produced and flowback waters from unconventional natural gas development of a sandstone formation.

From 2009 to 2011 the U.S. EPA conducted four sampling events meant to determine the presence (not extent) of groundwater contamination in the formation. In that study, DiGiulio et al. (2011) detected elevated concentrations of benzene, toluene, ethylbenzene, and xylenes (BTEX) in sampling wells at concentrations of 246, 617, 67, and 750 μg/L, respectively. Trimethylbenzenes and diesel range organics were detected at concentrations up to 105 and 4,050 μg/L, respectively, and total purgeable hydrocarbons were detected in the groundwater samples near the containment ponds (DiGiulio et al. 2011). Although these initial data indicated groundwater impacts that seem likely to be associated with unconventional gas production practices (U.S. EPA 2011a), the results of the study by DiGiulio et al. (2011) have been contested, and it is still unclear which part of the gas development process (if any) is responsible for the contamination. Further, there are geological differences between sandstone and shale, and fracturing is often conducted closer to the surface in sandstone formations. However, the findings suggest an association between water contamination and production activities that have also been identified in shale gas development (DiGiulio et al. 2011).

*Site discharge and improper waste disposal*. Fracturing fluids and produced waters can also contaminate underground sources of drinking water during waste management and disposal. Flowback and produced waters are often contained in evaporation ponds, pits, and tanks, in some cases in very close proximity to residences (Bamberger and Oswald 2012; Rozell and Reaven 2012). These containment ponds are often, but not always, lined to protect against leakage; however, case studies have documented reported ruptures to these liners that may have led to water and soil contamination and contributed to fish and livestock deaths (Bamberger and Oswald 2012). An analysis of waste obtained from reserve pits indicated the potential for exposure to technologically enhanced naturally occurring radioactive material and potential health effects from individual radionuclides (Rich and Crosby 2013).

Groundwater contamination can also result from surface spills at active well sites. Gross et al. (2013) analyzed data from the Colorado Oil and Gas Conservation Commission (http://cogcc.state.co.us) and noted 77 reported surface spills (associated with < 0.5% of active wells) impacting groundwater in Weld County, Colorado. The groundwater samples were analyzed for BTEX components. Most notably, benzene measurements exceeded the U.S. EPA National Drinking Water maximum contaminant level of 5 ppb in 90% of the samples (Gross et al. 2013). Because baseline-sampling measurements were not available, the background BTEX concentrations remain unclear. However, natural groundwater concentrations are typically low near deposits of crude oil, coal, and natural gas (Gross et al. 2013).

## Discussion

*Future research needs*. There is a growing body of scientific literature on the environmental public health dimensions of shale gas development; however, a number of important data gaps persist. Measurements of emissions and atmospheric concentrations should be conducted among diverse geographies, both indoors and outdoors, to help to estimate the types and magnitude of population exposures to pollutants associated with shale gas development. In addition, studies that take into account personal exposures and time–activity patterns of individuals would be helpful in assessing epidemiologically meaningful exposures. These studies could include the use of personal monitors and sampling of household drinking water in conjunction with health records to look at disease outcomes.

Perhaps the most important information gap is the lack of epidemiological studies. There is a need to assess the strength of the association between risk factors, such as air pollution and water contamination, and health outcomes among populations living in close proximity to shale gas develoment activities compared with those populations living in areas without these activities. Although lacking in definitive proof of cause and effect, self-reporting health surveys and environmental testing have suggested possible adverse health outcomes from shale gas development in Pennsylvania (Steinzor et al. 2013). Of particular interest are the epidemiological studies on vulnerable populations, including pregnant women, young children, the elderly, and those with compromised immune systems, who live, work, and play in close proximity to shale gas development. Because workers are likely to be the first and the most exposed demographic from shale gas development, further occupational health studies are also needed.

There have been some efforts in epidemiology and risk assessment, including a recent retrospective cohort study by that examined associations between maternal residential proximity to natural gas development and a number of birth outcomes. The authors found no positive association between density and proximity of wells within a 10-mile radius of maternal residence and prevalence of oral clefts, preterm birth, or term low birth weight. However, the researchers did observe a positive association between density and proximity of pregnant mothers to shale gas development and the prevalence of congenital heart defects and possibly neural tube defects in their newborns (McKenzie et al. 2014).

There have been some other epidemiological efforts as well, including a study funded by America’s Natural Gas Alliance that evaluated associations between childhood cancer incidence in Pennsylvania and hydraulic fracturing sites (Fryzek et al. 2013). The authors included 29,000 hydraulically fractured wells drilled between 1990 and 2009 in their analysis and obtained data on childhood cancers from the Pennsylvania cancer registry for this time period. However, shale gas development did not begin in Pennsylvania until 2006, when four wells of this type were drilled. In fact, only 726, or 2.5% of the 29,000 wells in their database, were relevant to directionally drilled shale gas wells. Unfortunately, this exposure misclassification and the disregard for the extended latency periods of many childhood cancers render this study inconclusive as to the effect of shale gas development on childhood cancer rates. The study by Fryzek et al. (2013) demonstrates the need for more epidemiological assessments that pay attention to the latency periods of environmentally mediated diseases.

Epidemiological investigations are challenged by the difficult task of identifying specific risk factors and the uncertainty in exposure classification because compounds used in shale gas development are often not disclosed. In these cases of uncertainty, a comprehensive water monitoring and—under certain circumstances, a biomonitoring program—that uses both targeted and nontargeted strategies would be useful. Useful data could be generated by targeted testing for specific compounds known to be associated with shale gas development in drinking water supplies and in the blood and urine of a representative sample of individuals living in close proximity to shale gas development. Nontargeted techniques, including time-of-flight mass spectrophotometers (TOF-MS), may also be helpful. Rather than monitoring for individual chemicals, TOF-MS has been important for the progress of biomonitoring in recent years by allowing researchers to monitor for tens of thousands of organic compounds at a time. This enables researchers to circumvent policy issues that do not require companies to disclose the compounds they employ in their activities, such as is the case in many regions throughout the United States.

Even with full disclosure of the chemicals added to fracturing fluid, the ability to link chemicals to specific health outcomes remains difficult. Fracturing fluids and flowback and produced wastewaters are complex mixtures of chemicals with individual and possibly cumulative and synergistic properties. Many health outcomes are not specific to chemicals associated with shale gas development (e.g., headaches can be caused by a number of factors, rashes can be nonspecific, and asthma can be induced through a number of pathways), complicating the task of assessing associations between exposures and health outcomes. In turn, more exposure assessments and water and air monitoring should be undertaken to investigate the full suite of compounds emitted to the environment from these activities.

The chemicals contained in fracturing fluids are often not publicly disclosed because of trade secret laws and exemptions under the Energy Policy Act of 2005 that further confound environmental public health research. Moreover, the U.S. EPA is precluded from regulating hydraulic fracturing under the Safe Drinking Water Act (1974), and Congress expressly exempted hydraulic fracturing from the Underground Injection Control program (U.S. EPA 2012b). The nondisclosure of these chemicals creates research barriers because it is difficult to monitor for unknown compounds.

*Limitations*. In this review, we focused on the peer-reviewed scientific literature on the environmental public health dimensions of shale gas development. Although we used a broad search strategy, some publications and other relevant data could have been missed in our literature searches. However, we consider this to be a substantive summary of the currently available literature. Results of future studies will clarify the scientific understanding of the environmental public health concerns of shale gas development.

## Conclusion

We reviewed the body of evidence of potential environmental public health dimensions of shale gas development. Scientific modeling and field investigations have helped to illuminate the emerging environmental issues with which shale gas production may be associated. Several studies have suggested that shale gas development contributes to pollutants in ambient air at concentrations known to be associated with increased risk of morbidity and mortality (Colborn et al. 2014; Kemball-Cook et al. 2010; McKenzie et al. 2012, 2014). Similarly, some evidence supports theories of water contamination risks through a variety of pathways, most notably during wastewater transport and disposal and through failed cement in wells with poor structural integrity (Vengosh et al. 2013; Vidic et al. 2013; Warner et al. 2013a). The existing peer-reviewed scientific data suggest that there are potential risks that could possibly influence public health. More research is needed to clarify the magnitude of these concerns. Because shale gas development activities have accelerated dramatically over the past decade, the need for well-designed empirical studies becomes increasingly apparent.
